# Ocular pathogenesis and immune reaction after intravitreal dispase injection in mice

**Published:** 2012-04-07

**Authors:** Juan Tan, Yaqin Liu, Wei Li, Qianying Gao

**Affiliations:** State Key Laboratory of Ophthalmology, Zhongshan Ophthalmic Center, Sun Yat-sen University, Guangzhou, China

## Abstract

**Purpose:**

The purpose of the current study was to examine the ocular pathogenesis and immune reaction in mice after intravitreal dispase injection.

**Methods:**

Three microliters of dispase at a concentration of 0.2 U/μl were injected into the vitreal cavities of 4–6-week-old mice. Hematoxylin and eosin staining, immunofluorescence analysis, and electroretinograms of the eyes were then performed to assess ocular changes, and enzyme-linked immunospot assays and intracellular staining of single-cell suspensions of the spleens were used to detect immune changes during an 8 week observation period.

**Results:**

Neutrophils were the main inflammatory infiltrating cells appearing at the anterior chamber. No cluster of differentiation (CD)3+ labeled T cells, F4/80+ labeled macrophages, or CD56+ labeled natural killer cells were found in the vitreal cavities or retinas in dispase-injected mice within 5 days after injection. Proliferative vitreoretinopathy (PVR)-like signs first appeared at 2 weeks, gradually increased thereafter, and reached peak values at 8 weeks. There was a statistically significant difference in b-wave amplitudes between the PVR and saline-control eyes. Enzyme-linked immunospot assays and intracellular staining showed that specific CD4+ and CD8+ labeled T cells were not involved in dispase-injected mice.

**Conclusions:**

Our data show that neutrophils in the anterior chamber and PVR-like signs in the retinas were found, and that specific immune reactions were not involved after intravitreal dispase injection in mice.

## Introduction

Dispase, a proteolytic enzyme able to harvest and culture cells due to its ability to cleave the basal membrane in various tissues, can be used as an intravitreal injection material to induce proliferative vitreoretinopathy (PVR) in the eyes of mice [1,2] and rabbits [3-9]. PVR is the most common cause of recurrent retinal detachment after retinal detachment repair, occurring in 5%–11% of patients [10,11]. Basic research has indicated that PVR is characterized by the formation of scar-like fibrous tissue containing myofibroblasts derived from transdifferentiated retinal pigment epithelial (RPE) cells and other cell types, such as glial cells, that have entered the vitreous cavity and induced the contraction of cellular membranes within the vitreous cavity on both detached retinal surfaces [12-14].

Dispase is a heterogeneous protein, and its intravitreal injection may cause some immune reaction and ocular change, not only in the vitreous and retina, but also in the anterior chamber. The autoimmune hypothesis has been prompted by the observation that a PVR-like disease can be induced in rabbits by immunization with the retinal autoantigens opsin, antigen S, and interphotoreceptor retinoid-binding protein [15]. Also apparently supporting the autoimmune hypothesis is the fact that PVR patients display signs of active immune processes in their epiretinal or subretinal membranes, vitreous cavities, subretinal fluids, and serum samples [16-22]. Similar signs of immune activation have been reported for the cells present in the vitreous cavities and subretinal fluids of PVR patients [19,23-26]. Sera of PVR patients have been reported to contain increased concentrations of S-antigen (S-Ag) [27] and S-Ag-specific autoantibodies [28]. The induction of these autoantibodies in particular implies that after iatrogenic eye injury, the exposure to S-Ag triggers an autoantigen-specific B cell response. Since autoantibody production of protein self-antigens is strictly dependent on cluster of differentiation (CD)4 T cell help [29], the presence of these antibodies also implies an autoimmune T cell response against these self-proteins. Thus, these autoreactive T and B cells could conceivably mediate the pathology underlying PVR. While the above findings are consistent with an autoimmune hypothesis for PVR, they do not prove it, and it has been difficult to establish whether the immune reactions seen represent the cause or merely an epiphenomenon of the disease [30].

Can dispase trigger autoreactive B or T cell response? To the best of our knowledge, the pathogenesis of the anterior chamber and immune reactions has not been well documented. Therefore, it is important to study the ocular pathogenesis and immune reaction after intravitreal dispase injection in mice. In the present study, we analyzed these outcomes after intravitreal dispase injection during an 8 week observation period.

## Methods

### Mice

Four- to six-week-old wild-type C57BL/6 mice were purchased from the South Medical University Animal Center (Guangzhou, China). Animal husbandry and experimental procedures were approved by the Animal Research Committee of the Zhongshan Ophthalmic Center, Sun Yat-sen University (Guangzhou, China). All animals were housed in a specific pathogen-free biohazard level-2 facility maintained by the Zhongshan Ophthalmic Center in accordance with the Association for Assessment and Accreditation of Laboratory Animal Care guidelines.

### In vivo model of proliferative vitreoretinopathy induced by dispase intravitreal injection

The murine PVR model was induced by dispase (Gibco, Tokyo, Japan), as previously described [1,2]. Intravitreal injections were performed in the dorsonasal quadrant (1 o’clock) 1.5 mm away from the corneal limbus of the right eye. Three μl of dispase at a concentration of 0.2 U/μl were injected into the vitreal cavities using a Hamilton syringe fitted with a 30 G needle. Control animals received 3 μl of sterile saline solution. Female mice, 4–6 weeks old, were anesthetized with 4.3% chloral hydrate (0.01 ml/g; Zhongshan Ophthalmic Center, Sun Yat-sen University, Guangzhou, China). Pupils were dilated with 0.5% tropicamide (Shenyang Sinqi Pharmaceutical Co., Ltd). Intravitreal injections were performed in the dorsonasal quadrant (1 o’clock) 1.5 mm away from the corneal limbus of the right eye. Three μl of dispase at a concentration of 0.02 U/μl were injected into the vitreal cavities using a Hamilton syringe fitted with a 30 G needle. Control animals received 3 μl of sterile saline solution. All experimental procedures adhered to the guidelines of the Association for Research in Vision and Ophthalmology Statement for the Use of Animals in Ophthalmic and Vision Research.

### Experimental groups

The mice were divided into a dispase-injected group and a saline-injected control group (n=92, n=68). These two groups were then equally divided into seven subgroups (n=6, n=4) at the 0 h, 4 h, 8 h, 12 h, 24 h, 48 h, and 5 day time points and five subgroups (n=10, n=8) at the 1, 2, 4, 6, and 8 week time points.

### Follow-up examinations and the development of proliferative vitreoretinopathy

The injected eyes were examined and assessed—including the cornea, lens opacity, intravitreal hemorrhages, and the fundus—using a surgical microscope at each time point after intravitreal injection. Because intravitreal hemorrhages and cataracts have often occurred in various studies [31], clinical PVR-like signs were defined as the presence of one of the following three symptoms: retinal folds, epiretinal membranes, and an uneven iris at 1, 2, 4, 6, and 8 weeks during this experiment. This evaluation system was modified from Cantó Soler MV [1,2].

### Tissue preparation and histological and immunofluorescence analysis

Mice used for histological studies were sacrificed at 0 h, 4 h, 8 h, 12 h, 24 h, 48 h, and 5 days or 1, 2, 4, 6, or 8 weeks after injection, and dissected eyes were cryopreserved using the optimal cutting temperature (Sakura Finetechnical, Torrance, CA) compound. For immunofluorescence analysis and hematoxylin and eosin (H&E) staining, consecutive 6 μm thick sections of each sample were cut and thaw-mounted onto poly-L-lysine-coated glass slides. For confocal microscopy, double immunostaining was performed using two primary antibodies incubated respectively for about 20 h at room temperature and then incubated with secondary antibodies for about 1 h in the dark. Primary antibodies served as markers, including glial acidic fibrillary protein (GFAP), glutamine synthase (GS), retinal pigment epithelium 65 (RPE65), alpha smooth muscle actin (a-SMA), F4/80, anti-CD3, and anti-CD56. Dilutions and the source of each primary antibody are described in Table 1. Negative controls were made by omitting the primary antibodies. Three secondary antibodies were used in this study: R-phycoerythrin-conjugated goat antirat IgG (1:10; Southern Biotechnology Associates, Inc., Birmingham, AL), R-phycoerythrin-conjugated goat antimouse IgG (1:10; Southern Biotechnology Associates, Inc.), and fluorescein isothiocyanate–labeled goat antirabbit IgG (1:10; KPL, Gaithersburg, MD). Sections were washed four times in PBS (5 min each time) and mounted under coverslips in antifade solution (Applygen Technologies Inc., Beijing, China) for observation with a fluorescence microscope (Carl Zeiss Far East Co. Ltd., HK).

**Table 1:** Characteristics of primary antibodies used in this study.

**Antigen**	**Antibody class**	**Source**	**Dilution**
RPE65	Mouse monoclonal	Abcam plc 332 Cambridge Science Park, Cambridge, UK	1:100
GFAP	Mouse monoclonal	Abcam plc 332 Cambridge Science Park, Cambridge, UK	1:500
GS	Rabbit polyclonal	Abcam plc 332 Cambridge Science Park, Cambridge, UK	1:50
a-SMA	Rabbit polyclonal	Abcam plc 332 Cambridge Science Park, Cambridge, UK	1:100
CD3	Rat monoclonal	R&D Systems, Inc, USA	1:50
CD56	Rabbit polyclonal	Boster Biologic Technology, WuHan, China	1:100
F4/80	Rat monoclonal	Abcam plc 332 Cambridge Science Park, Cambridge, UK	1:10

### Electroretinograms

Electroretinograms (ERGs) of dispase-injected PVR mice (n=14) and saline-injected control mice (n=14) were performed at 6 and 8 weeks in a full-field dome, using methods similar to those used in the clinic and stimuli comparable to those specified by the International Society for Clinical Electrophysiology of Vision [32]. The eyes were dilated with 0.5% tropicamide and dark-adapted for at least 30 min. Mice were anesthetized with a saline solution containing ketamine hydrochloride (30 mg/kg) and chlorpromazine hydrochloride (15 mg/kg). A gold-wire coil placed on one cornea was referenced to a needle electrode in the scalp. A needle electrode in the tail served as ground. The recordings were made using the Roland Ganzfeld system and PC-based signal acquisition and analysis software (Fa. Roland Consult, Brandeburg, Germany). Scotopic, mesopic, photopic, and oscillatory potentials, as well as 30 Hz flicker ERG responses were recorded. The bright-flash response was elicited using the ISCEV standard flash of 2.4 cd/m2 (settings: −25dB flash, fix on, backlight off 0.5HZ). After light adaptation of 10 min with a steady background illumination of 10 cd/m^2^, photopic responses (settings: 0dB standard flash, fix on, backlight off 0.2HZ) and 30 Hz flicker ERGs were recorded. In this way, the a- and b-wave amplitudes and the implicit times of the standard responses were determined [33].

### Enzyme-linked immunospot assay

Mice were sacrificed at 1, 2, 4, 6, or 8 weeks after injection. An enzyme-linked immunospot (Enzyme-Linked ImmunoSpot assay [ELISpot], Cellular Technology Ltd, Cleveland, OH) assay was performed, as described by Jager et al. [34]. In brief, single-cell suspensions were prepared from the spleens of individual mice. The immunospot plates (MAHAS45; Millipore, Bedford, MA) had the regular format of 96 wells. Ethanol (70%) was added at 15 μl per well for prewetting, and the plates were washed three times with PBS. After washing, the plates were coated with antimouse-interferon (IFN)-γ capture Ab (BD, San Diego, CA) at 5 μg/ml in PBS and antimouse-interleukin (IL)-2 capture Ab (BD) at 5 μg/ml in PBS, respectively; they were then stored overnight at 4 °C. The plates were then blocked with Roswell Park Memorial Institute (RPMI) 1640 medium for 2 h at room temperature and washed three times with PBS. The single-cell suspensions were plated in complete RPMI 1640 and added at 1×105 cells per well to the 96-well plates. In each well, dispase (Gibco, Tokyo, Japan) was added as a stimulus at 0.02 U/ml; as a positive control, 2 μg/ml phytohemagglutinin (PHA; Fluka, Sigma, Carlsbad, CA) was added in the absence of dispase. Finally, as a negative control, PBS was added in the absence of dispase. After 20 h incubation at 37 °C in CO_2_ incubators and washing, 2 μg/ml of biotinylated antimouse-IFN-γ detection Ab (BD) in PBS/BSA (BSA)/Tween (10 g/l BSA with 0.5% Tween) and 2 μg/ml antimouse-IL-2 detection Ab (BD) in PBS/BSA/Tween (10 g/l BSA with 0.5% Tween) were added. After 2 h incubation at room temperature and washing three times with PBS/Tween, 100 uL/well of streptavidin- horseradish peroxidase (HRP; 1:100 dilution; BD) in PBS/BSA/Tween was added for 1 h at room temperature and then washed with PBS/Tween, followed by PBS. The 3-amino-9-ethylcarba (AEC) working solution was freshly prepared by mixing 9.8 ml AEC substrate solution (BD) with 200 μl AEC chromogen solution (BD); 100 ul of the mixture was plated per well. The plates were developed for 15–20 min, after which the reaction was stopped by rinsing with tap water. The plates were air dried overnight and read by the Champ Spot II ELISpot reader (Sage Creation, Beijing, China). The indicated spot numbers per well represent the mean values of three replicates.

### Intracellular staining and flow cytometry

Intracellular staining and a flow cytometry assay were performed, as previously described [35]. Single-cell suspensions from the spleens of model mice at 4 weeks were prepared. Dispase was added as a stimulus at 0.02 U/ml; as a positive control, 2 μg/ml PHA (Fluka; Sigma, Louis, MO) were added in the absence of dispase. As a negative control, PBS was added in the absence of dispase. After 1 h of incubation, Brefeldin A (Sigma) at a final concentration of 10 μg/ml was added to cultures to enable intracellular protein to accumulate in all stimulations. After incubation for a total of 5 h, the cells were harvested, washed twice with PBS, fixed with 4% paraformaldehyde, and permeabilized with PBS containing 0.1% saponin (Fluka) plus 0.5% BSA buffer overnight at 4 °C. They were then stained with anti-CD4, anti-CD8, anti-IFN-γ, or isotype control Abs (BD) for 30 min at 4 °C and washed twice with PBS before being resuspended in PBS containing 0.5% BSA and 0.1% NaN_3_. Cells were acquired using a FACSCalibur flow cytometer (BD Biosciences, San Diego, CA), and FACS data were analyzed using FlowJo (BD Biosciences). Isotype-matched controls for cytokines were included in each staining.

### Data and statistical analysis

Results are expressed as mean±standard deviation (SD). ERG data were compared using the Student *t* test. A value of p<0.05 was considered statistically significant.

## Results

### Early inflammatory infiltration (occurring at 0, 4, 8, 12, 24, and 48 h, and 5 days)

**Figure 1:**
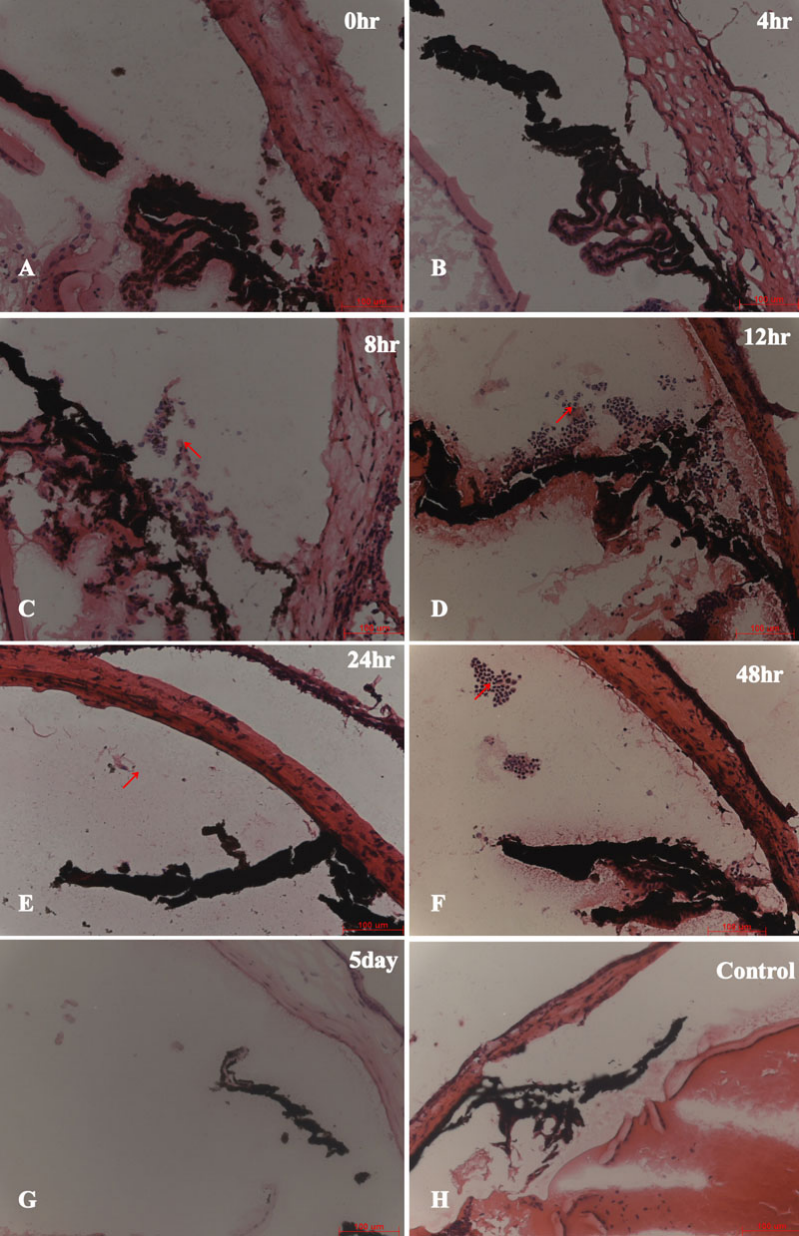
Early inflammatory infiltration profile (0–5 day time points) in diapase-injected and saline-injected control mice. Panels **A**-**G** shows inflammatory infiltration profile 0–5 days. Panel **H** shows a control. Neutrophils appeared from hour 8 (**C**) to hour 48 (**F**) in the anterior chamber of dispase-injected eyes but not saline-injected eyes (hematoxylin and eosin [H&E] staining, scale bar 100 μm). All the arrows of panels **C**-**F** shows neutrophils.

**Figure 2:**
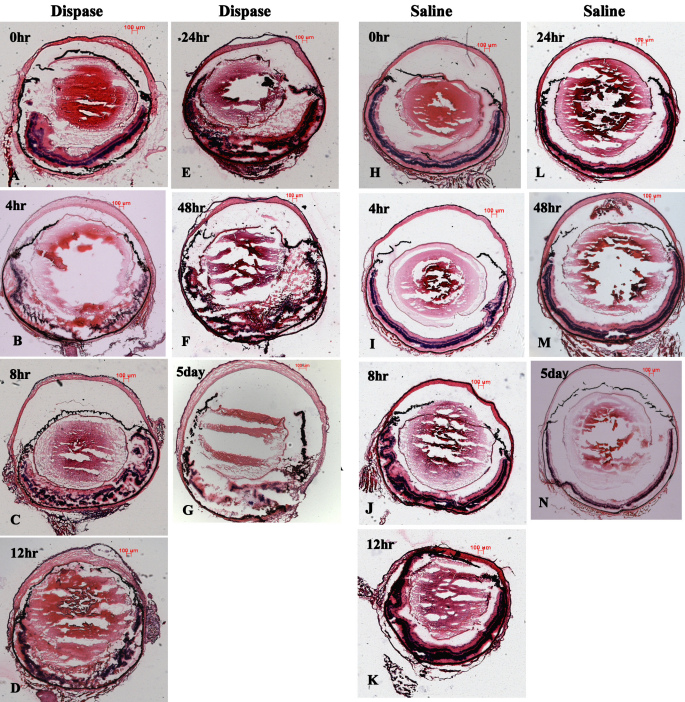
Ocular morphology during early inflammatory infiltration phase showed retinal structure damaged, in diapase-injected eyes but relatively intact in saline-injected eyes (H&E staining, scale bar 100 μm; **A**-**K**).

**Figure 3:**
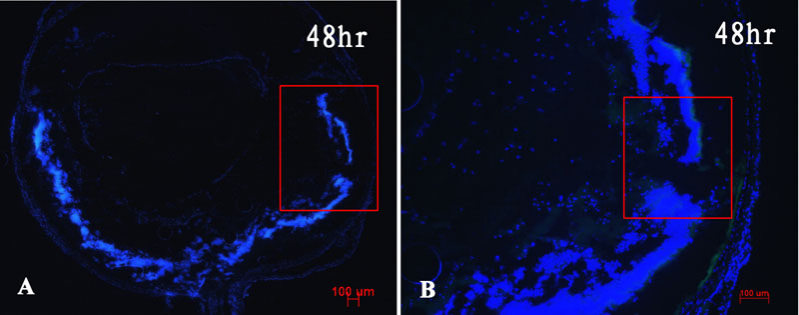
Immunofluorescence analysis showed no cluster of differentiation (CD)3+ labeled T cells, F4/80+ labeled macrophages, or CD56+ labeled natural killer (NK) cells involved in dispase-injected eyes at 48 h time point (**A**, **B**). Bluish cells stained with Hoechst 33342 (scale bar 100 μm).

In the dispase-injected group, the results of H&E-stained sections of the model eyes showed early inflammatory infiltration after dispase injection at 0, 4, 8, 12, 24, and 48 h and 5 days. Neutrophils were the main inflammatory infiltrating cells appearing at the anterior chamber (Figure 1) instead of the vitreous cavity (Figure 2) during early inflammatory infiltration. The neutrophils appeared from hour 8 to hour 48 at the anterior chamber, as shown in Figure 1 and Figure 2. However, in the saline-injected control groups, the H&E-stained sections exhibited normal eye morphology, without inflammatory infiltration cells, at all time points. No CD3+ labeled T cells, F4/80+ labeled macrophages, or CD56+ labeled natural killer (NK) cells were observed in the vitreous cavities or retinas at any early time point in either of the two groups (Figure 3).

### Proliferative vitreoretinopathy development at 1, 2, 4, 6, and 8 weeks

**Figure 4:**
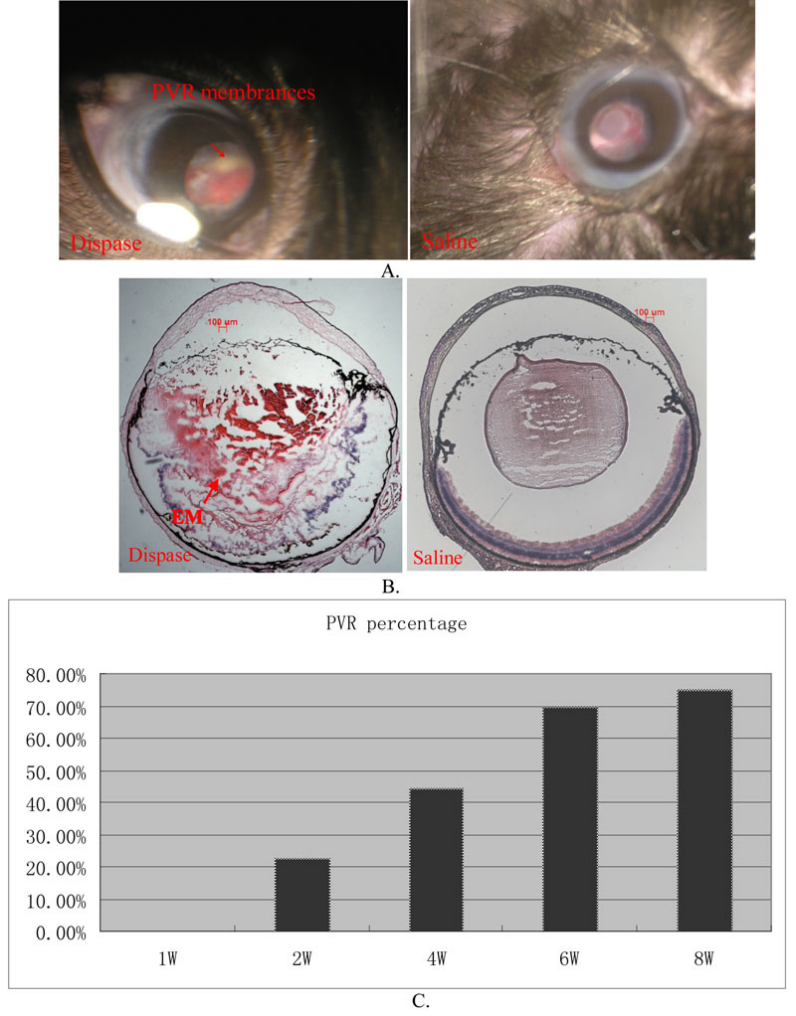
Dispase-injected mice developed cardinal features of proliferative vitreoretinopathy (PVR). **A**: Ocular fundi of dispase-injected eye and saline-injected control eye. Arrow shows PVR membranes. **B**: Hematoxylin and eosin (H&E) staining of dispase-injected PVR eye and saline-injected control eye. Arrow shows proliferative epiretinal membranes (EM). **C**: PVR percentages at 1, 2, 4, 6, and 8-week time points after dispase injection.

**Figure 5:**
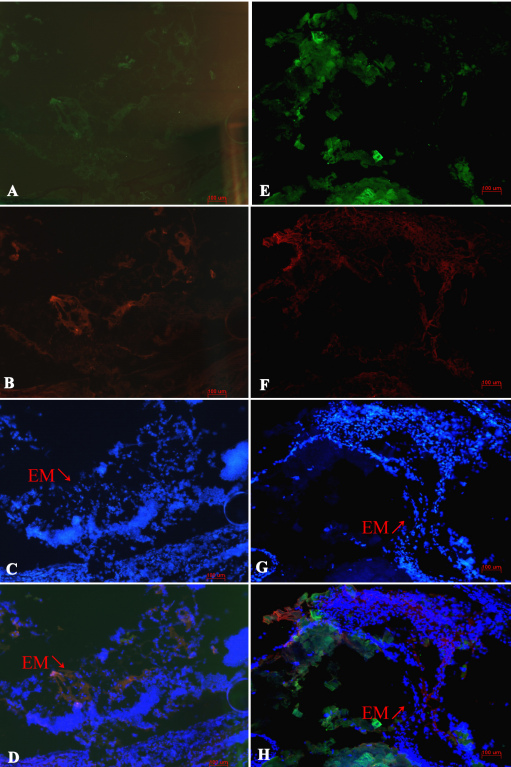
Immunofluorescence analysis showed alpha smooth muscle actin (α-SMA; **A**), glial acidic fibrillary protein (GFAP; **B**), glutamine synthase (GS; **E**), and retinal pigment epithelia Protein 65 (RPE-65; **F**) in epiretinal membranes (EMs) of proliferative vitreoretinopathy (PVR) model eyes, indicating fibroblast cells, Müller cells, astroglial cells, and RPE cells involved in the process of PVR. Hoechst 33342 for nucleic acid stained alone (**C**, **G**). **D** is the merged picture of **A**-**C**, **H** the merged picture of **E**-**G** (a triple staining). Arrow shows EM.

After dispase injection, hemorrhages, cataracts, and PVR were observed in the dispase-injected groups (Figure 4). PVR-like signs first appeared at 2 weeks; subsequently, they gradually increased and reached their peak values at 8 weeks. The PVR percentages at 1, 2, 4, 6, and 8 weeks were 0%, 23%, 44%, 70%, and 75%, respectively, in the dispase-injected groups. H&E-stained frozen sections clearly showed marked proliferative membranes, retinal detachment, serous fluid between the RPE and the sensory retinas, and destructed retinas and lenses. In the process of PVR development, the RPE65, GFAP, GS, and a-SMA labeled cells were involved in the PVR eyes of mice (Figure 5). No structural abnormalities were found in the control eyes at the same time points.

### Electroretinogram results

**Figure 6:**
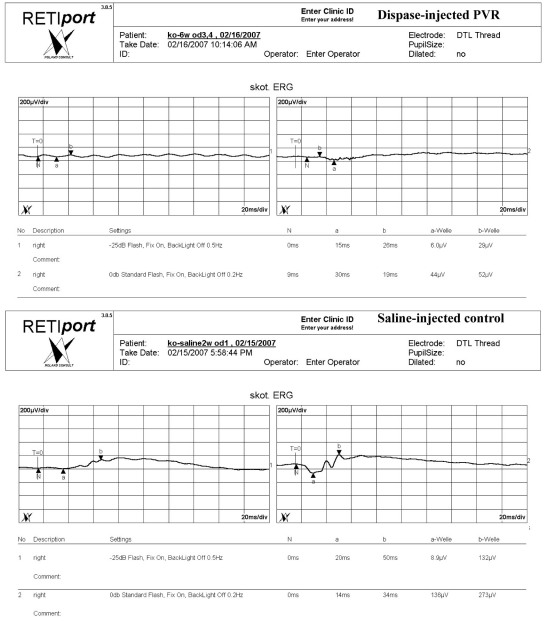
Electroretinograms (ERG) of the dispase-injected proliferative vitreoretinopathy (PVR) model eyes and saline-injected control eyes. Scotopic ERGs showed significant decreases in b-wave amplitudes, but no decreases in a-wave amplitudes of dispase-injected PVR eyes compared with saline-injected control eyes.

As shown in Figure 6 and Table 2, mesopic ERG (0dB standard flash, fix on, backlight off 0.2HZ) data showed decreases in the amplitude of the a-wave in dispase-injected PVR eyes, suggesting photoreceptor dysfunction, as well as reductions in b-wave amplitudes, indicating that retinal interneurons (bipolar cell function) were affected as well. However, scotopic ERGs (−25dB flash, fix on, backlight off 0.5HZ) also showed significant decreases in the b-wave amplitudes of dispase-injected PVR eyes, but no decreases in the a-wave amplitudes of dispase-injected PVR eyes, indicating that rod cell function was partially preserved.

**Table 2:** ERG data in the dispase-injected PVR and saline-control eyes.

**Scotopic ERG (−25dB Flash, Fix On, BackLight Off 0.5HZ)**			
		**amplitude**	**p-value**
a-wave	saline-injected control eyes	10.817±4.6006/uV	0.2120(>0.05)
	dispase-injected PVR eyes	8.0143±4.3735/uV	
b-wave	saline-injected control eyes	100.00±29.698/uV	0.001(<0.05)
	dispase-injected PVR eyes	19.886±7.774/uV	
**Mesopic ERG (0dB Standard Flash, Fix On, BackLight Off 0.2HZ)**			
a-wave	saline-injected control eyes	46.167±27.766/uV	0.0110(<0.05)
	dispase-injected PVR eyes	21.836±11.487/uV	
b-wave	saline-injected control eyes	180.50±54.099/uV	0.0013(<0.05)
	dispase-injected PVR eyes	48.57±24.747/uV	

### Enzyme-linked immunospot assay

**Figure 7:**
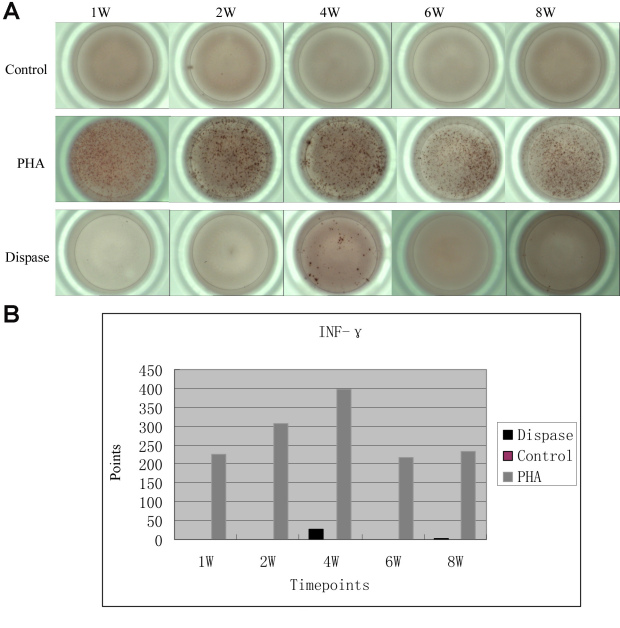
Enzyme-Linked ImmunoSpot (ELISpot) assay of interferon (INF) -γ in single T cell suspensions from dispase-injected and saline-injected mouse spleens. **A**: Compared with positive control group (stimulated with phytohemagglutinin, PHA), IFN-γ values were only detected at 4-week and 8-week time points in dispase-injected mice, and not detected at any time point in saline-injected mice. **B**: In the graph of counting statistics, there were only 25.5 points at the 4-week time point, and 2.5 points in the 8-week for dispase-injected mice.

At the 1, 2, 4, 6, and 8 week time points, IFN-γ values were only detected in the single-cell suspensions of the T cells of dispase-injected mice at 4 and 8 weeks. However, when compared with those in the positive control (stimulated with PHA), there was no immune reaction in dispase-injected mice or in saline-injected mice (Figure 7). IL-2 was not detected at 1, 2, 4, 6, or 8 weeks in either dispase-injected mice or saline-injected mice.

### Intracellular staining

**Figure 8:**
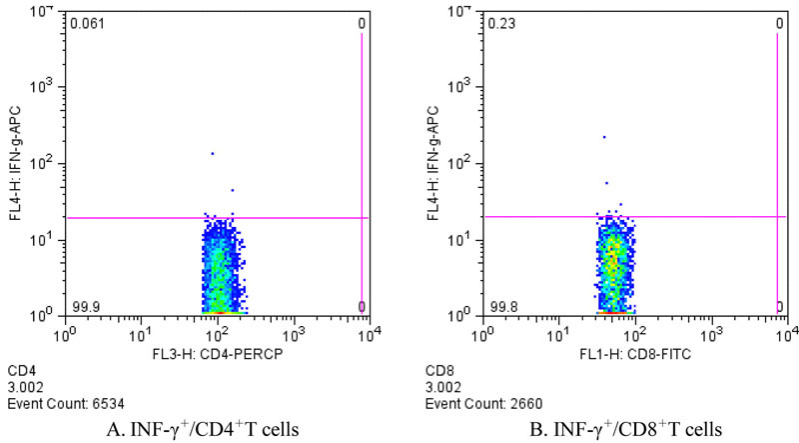
Intracellular staining at the 4-week time point in single T cell suspensions from dispase-injected PVR-model mouse spleens. **A**: IFN-γ+/CD4+T cells 0.061% of the total CD4+T cells. **B**: IFN-γ+/CD8+T cells 0.23% of the total CD8+T cells.

To test if IFN-γ was secreted from the spleens of the dispase-injected mice at 4 weeks, intracellular staining was performed. As shown in Figure 8, there were IFN-γ+/CD4+ (0.061%) or IFN-γ+/CD8+ (0.23%) T cells in the single-cell suspensions from the spleens of dispase-injected mice at 4 weeks. The above results indicate that IFN-γ was not secreted by CD4+ or CD8+ labeled T cells in dispase-injected mice.

## Discussion

Recently, there has been an increasing trend to induce the PVR model using dispase [1-9]. In our study, we successfully replicated the murine PVR model and observed the ocular pathogenesis and immune reaction after intravitreal injection of dispase. This was the first demonstration indicating that neutrophil cells appeared in the anterior chamber of eyes and that specific immune reactions were not involved after intravitreal dispase injection in mice.

PVR can be induced via intravitreal injection of different revulsants, such as RPE cells [36,37], macrophages [38,39], and fibroblasts [40]. Compared with these revulsants, dispase is easily available and does not lead to exogenous cell development in the PVR model. Hence, in the mouse PVR model, dispase induced a PVR-like condition with a strong contribution of macrophage- and glial-derived cells [1]. Regarding the rabbit PVR model, several authors have reported effects of matrix metalloproteinases [4] and a DNA-RNA chimeric ribozyme-targeted proliferating cell nuclear antigen [5]. Researchers have also reported Müller glial cells displaying upregulation of purinergic P2 receptor–mediated responses [6,7] and the interaction of cell surface molecules, extracellular matrix proteins, cytoskeletal elements [8], and a foldable capsular vitreous body [9] in PVR. However, due to complications such as cataracts and lens dissolution after dispase injection, the eye fundi of mice have been impossible to observe via microscope in this and other studies [31]. Therefore, we modified the PVR development from the five scores used by Suburo’s group [1] to three scores. Once cataracts and uneven irises occur, tractional retinal detachment is inevitable in model eyes. We confirmed this result based on the H&E and immunofluorescence analysis of the frozen sections.

Regarding the early stage of PVR, we first reported that neutrophils were the main inflammatory infiltrating cells appearing at the anterior chamber instead of the vitreous cavity during early inflammatory infiltration (i.e., before 5 days), as shown in Figure 1, Figure 2 and Figure 3. One explanation for these infiltrating cells during the time between 8 h and 48 h after dispase injection might be a break of the blood vessel integrity near the ciliary body caused by dispase itself or methodological variations. These neutrophils might represent separate events in the etiology of PVR. In addition, no CD3+ labeled T cells, F4/80+ labeled macrophages, or CD56+ labeled NK cells appeared in the vitreous cavities or retinas before 5 days. This is likely due to the strong digestive functions of the dispase enzyme.

The role of inflammatory and immune cells in PVR membranes in the late stage of PVR has been reported in several studies [1,18,26,41]. Suburo’s group showed that CD3 immunoreactivity was not detected in saline- or dispase-injected eyes [1]. T lymphocytes were found in five of the eight subretinal membranes. CD4+ T cells were demonstrated in four of the membranes and CD8+ T cells in one of the membranes. T cells bearing the IL-2 receptor were found in two of four membranes studied. Macrophages were found in four membranes. No B lymphocytes or neutrophils were observed, and there were no significant deposits of complement or immunoglobulins [18]. The findings of Canataroglu et al. suggest that IL-6 and IL-8 may be involved in the pathogenesis of proliferative diabetic retinopathy (PDR), PVR, and traumatic PVR. Cytological examination of the vitreous specimens revealed a predominance of macrophages (50%) in the PDR samples and a predominance of RPE cells (60%) in the PVR samples. In contrast, neutrophils predominated (88%) in the traumatic PVR samples [26]. Using the monoclonal antibodies EBM11 (pan macrophage) and 27E10 (early inflammatory stage marker), Esser et al. [41] observed that macrophages were predominantly found in traumatic PVR. Inflammatory stage macrophages could not be detected in PVR following rhegmatogenous retinal detachment and idiopathic macular pucker. In a previous study, T lymphocytes were found in 18 of the 21 PVR specimens and generally constituted a small percentage of the total cell number. CD4+ T cells were found in 14 of the 18 membranes containing T cells. Three of six frozen membranes contained T cells that were positive for the IL-2 receptor. Cells positive for the macrophage/monocyte marker were found in five of the 16 membranes studied. No B lymphocytes or neutrophils were observed, and there were no deposits of complement or immunoglobulins [42].

Our observations yielded similar results; that is, the intravitreal dispase injection induced PVR-like signs. Clinically significant PVR with retinal detachment developed in 75% of mice at 8 weeks, which is very similar to the 75% of mice [1] and 76% of rabbits [4] previously reported. In our study, for confocal microscopy, double immunostaining for RPE65, GFAP, GS, and a-SMA labeled cells was involved in the development of PVR, which is very similar to other studies [1,43]. GFAP is mainly expressed by astrocytes in the normal retinas of several species. Müller cells’ staining for GFAP in normal retinas is very faint. However, in pathologically changed retinas, Müller cells become immunopositive for GFAP in retinal detachment and PVR. GS is a marker for astrocytes. In retinas, many astrocytes with contact to ganglion cells also express GS. A-SMA is expressed by myofibroblasts. However, transdifferentiated RPE cells or macroglial cells (astrocytes as well as Müller cells) in epiretinal membranes of PVR eyes can also express this molecule. The ERG results further confirmed that the retinal functions of mice had been significantly damaged.

Our results regarding the ELISpot assay and intracellular staining first showed that specific immune systems do not play a major role in the pathogenesis of PVR. Although a great deal of information supports the role of the immune system in the development of PVR, the role of the immune system in the pathogenesis of PVR is still unclear. Data are sometimes contradictory, and it is difficult to establish whether the immune alterations seen in PVR are causes or consequences [30]. The results of a recent study did not support the assumption that CD44 has a functional role in the pathogenesis of PVR [8]. Although our ELISpot assay showed that INF-γ was detected in the single-cell suspensions of the T cells of dispase-injected mice at 4 and 8 weeks, when compared with those in the positive control (stimulated with PHA), there were no immune reactions in dispase- or in saline-injected mice (Figure 7). Interestingly, there were few IFN-γ+/CD4+ or IFN-γ+/CD8+ T cells determined by intracellular staining at a maximum of 4 weeks in dispase-injected mice. Thus, the IFN-γ found was not secreted by CD4+ or CD8+ T cells in these mice. It was concluded from these results that specific immune systems do not play a major role in the murine dispase-induced PVR model.

When compared with human PVR diseases, the murine dispase-induced PVR models expressed cardinal features of PVR in this study. Our data clearly showed that specific immune systems were not involved in the development of PVR. Despite the prevalence of the autoimmune hypothesis for PVR, the present treatment of human PVR in clinics targets inflammation and proliferation. Triamcinolone acetonide is being used as an anti-inflammatory drug for early-stage treatment, and 5-Fluorouracil (5-FU) is being used as an antiproliferation drug in advanced-stage treatment [44,45]. Therefore, dispase-induced murine PVR indeed models the human disease and can help us select drugs to treat PVR successfully.

In conclusion, our data show that neutrophils in the anterior chamber and PVR-like signs in the retinas were found and that specific immune reactions were not involved after intravitreal dispase injection in mice.
